# The association between workplace smoking bans and self-perceived, work-related stress among smoking workers

**DOI:** 10.1186/1471-2458-12-123

**Published:** 2012-02-13

**Authors:** Sunday Azagba, Mesbah F Sharaf

**Affiliations:** 1 Department of Economics, Concordia University, 1455 de Maisonneuve Blvd. West, Montréal, Quebec H3G 1M8, Canada

**Keywords:** Smoking bans, Stress, Unintended consequences, Fixed-effects

## Abstract

**Background:**

There is substantial empirical evidence on the benefits of smoking bans; however, the unintended consequences of this anti-smoking measure have received little attention. This paper examines whether workplace smoking bans (WSB's) are associated with higher self-perceived, work-related stress among smoking workers.

**Methods:**

A longitudinal representative sample of 3,237 individuals from the Canadian National Population Health Survey from 2000 to 2008 is used. Work-related stress is derived from a 12-item job questionnaire. Two categories of WSB's, full and partial, are included in the analysis, with no ban being the reference category. Analysis also controls for individual socio-demographic characteristics, health status, provincial and occupational fixed-effects. We use fixed-effects linear regression to control for individual time-invariant confounders, both measured and unmeasured, which can affect the relationship between WSB's and work-related stress. To examine the heterogeneous effects of WSB's, the analysis is stratified by gender and age. We check the robustness of our results by re-estimating the baseline specification with the addition of different control variables and a separate analysis for non-smokers.

**Results:**

Multivariate analysis reveals a positive and statistically significant association between full *(β = 0.75, CI = 0.19-1.32) *or partial *(β = 0.69, CI = 0.12-1.26) *WSB's, and the level of self-perceived, work-related stress among smoking workers compared to those with no WSB. We also find that this association varies by gender and age. In particular, WSB's are significantly associated with higher work stress only for males and young adults (aged 18-40). No statistically significant association is found between WSB's and the level of self-perceived work-related stress among non-smoking workers.

**Conclusion:**

The results of this study do not imply that WSB's are the main determinant of self-perceived, work-related stress among smokers but provides suggestive evidence that these may be positively related.

## Background

Tobacco use, including passive smoking, is a key risk factor for several illnesses such as cancer, heart attacks, cardiovascular disease, strokes [1] and is responsible for about 6 million deaths each year [2]. Parallel to the increased global awareness of the health risk of environmental tobacco smoke (ETS), public smoking bans, including workplace smoke-free policies, have become widespread in many countries. While these bans are primarily intended to protect non-smokers from the adverse effects of ETS, some empirical evidence suggests that they also encourage smoking cessation behaviors [e.g. [3-6]]. For example, Moher et al. [7] find consistent evidence that workplace smoke-free policies reduce cigarette consumption during the working day and exposure to ETS at work among non-smokers. In a meta-analysis of 26 studies, Fichtenberg and Glantz [4] find that workplace smoking bans (WSB's) decrease smoking prevalence and daily smoking intensity as well as exposure to ETS.

While there is a large and growing literature regarding the effects of WSB's on smoking behavior, their unintended effects have received little attention. Identifying these effects will be of importance to public policy, particularly since negative effects may limit the effectiveness of WSB's. There is evidence that public smoking bans including WSB's may lead to compensating smoking behavior, such as increased smoking during work breaks [8] and displaced smoking [9,10]. Adda and Cornaglia [10] find that in some instances, bans may increase exposure to ETS due to smokers switching from smoking in public areas where bans are enforceable to non-enforceable private areas. WSB's may increase tension between non-smoking and smoking workers [11]. In a UK study, Anderson et al. [12] find that WSB's in hospitals make nurses practice some dangerous smoking behaviors like stubbing out cigarettes in bins that contained paper towels and smoking in unsafe places. Moore et al. [13] find that a California smoking restriction in bars may increase exposure to ETS and other adverse effects. Particularly for women, the authors find that compliance with WSB's by smoking outside bars can constitute a threat to their physical safety and public image. In a related study, Adams and Cotti [14] observe an increase in accidents due to drunk driving because individuals drive long distances to bars with no smoking restrictions. WSB's may also lead to the accumulation of smoking trash, like cigarette butts and dead matches at the entrance to a workplace. This could increase the risk of fire and impose a cost on local governments to collect the trash. Also, a congregation of smokers at the entrance of a workplace increases exposure to ETS to people entering and leaving the workplace.

Workplace smoke-free laws may be linked to work-related stress in two ways. First, stress may result from both psychological and physical dependence on nicotine. This could be caused by a reduced level of nicotine in the blood arising from a requirement to restrict smoking during the working day. Studies have identified several symptoms of nicotine withdrawal, all of which may cause higher stress. These include anxiety, tension, depression and difficulty concentrating [15-17]. Second, smokers with severe self-control problems may be more vulnerable to high work-related stress especially if there is a full smoking restriction in the workplace.

The objective of this paper is to examine whether WSB's are associated with higher self-perceived work-related stress among working smokers, using longitudinal data from the Canadian National Population Health Survey (NPHS).

### Data

The NPHS is a nationally representative longitudinal survey of the Canadian population. The NPHS is based on a multistage stratified random sampling design. The survey started in 1994 and undertook a follow up of the same individuals every two years thereafter [18]. The NPHS excludes people living on Indian Reserves and Crown Lands, full-time members of the Canadian Forces and some remote areas of Ontario and Quebec. The data set contains a large number of variables related to health, as well as corresponding economic and socio-demographic variables. This study uses five waves (2000, 2002, 2004, 2006, and 2008) of the NPHS since the outcome variable "workplace stress" is not available for both the 1996 and 1998 waves. The attrition rates between two consecutive waves are: 7.6% (between wave 2000 to wave 2002), 7.5% (2002-2004), 5.4% (2004-2006) and 9.2% (2006-2008). In each wave, the NPHS sampling weights are adjusted in order for the data to be representative of the Canadian population. Accordingly, in this study, all analysis is population weighted using the NPHS sampling weights. To achieve the objective of this study, the analysis focuses mainly on smokers aged 18-65 years (n = 3,470). Smoking status is based on whether an individual at the time of the interview smokes or not. Detailed analysis of smoking prevalence in Canada using the NPHS is provided elsewhere [19]. After excluding missing information on the main outcome variables or other control variables, the unbalanced panel sample includes of 3,237 individuals and 7,763 person-year observations.

### Workplace stress

The Workplace Stress Index is a comprehensive measure of work-related stress that is derived from a 12-item job questionnaire (JCQ) by Statistics Canada; higher values indicate greater work stress. In each wave, employed respondents aged 15 and over are asked to evaluate their work situation based on multiple job-related questions. Individual's responses reflect perception about various dimensions of their work including: job security, social support, monotony, physical effort required and the degree of physiological demands and decision-making authority of each individual's job. Detail description of the outcome measure, work-related stress, has been extensively documented elsewhere [20-23]. Internal consistency of two sub-components of the NPHS JCQ, psychological demand (α = 0.34) and job control (α = 0.61) for the initial cross-sectional sample (1994/1995) has been reported [24]. Low or moderate internal consistency does not necessarily imply lack of validity of the JCQ, as it may well represent lack of redundancy in each item's contribution to the measurement of workplace-related stress [25]. For example, low internal consistency is plausible "where a measure records the inputs or the cause of the variable to be measured such as using life events to measure stress" [[25], pg. [44]].

### Workplace smoking bans

The main variable of interest in this study is WSB's. Three categories of WSB's are derived from the question: "At your place of work what are the restrictions on smoking?" Individuals' answers are coded as: (1) restricted completely; (2) allowed in designated areas; (3) restricted only in certain places; and (4) not restricted at all. These categories are reduced to three by combining (2) and (3), and the resulting three categories are designated: full ban, partial ban and no ban respectively, no ban being the reference category. The analysis also controls for other variables. Socio-demographic characteristics are represented by: age, marital status, educational attainment, ethnicity (measured by language ability), work status (full time or part time), income level and drinking status. Health status is captured by the number of individual chronic diseases and health utility index (HUI). The HUI is a set of generic, preference-based systems for measuring health status developed by the health utilities group at McMaster University [26]. Provincial and occupational dummies are also included in the analysis, in order to account for unobserved location differences in working condition between provinces and individual occupation-specific characteristics. The complete list of explanatory variables and their descriptive statistics are presented in Table 1. Access to the longitudinal version of the NPHS data set requires authorization by Statistics Canada. The first author was granted access by Statistics Canada.

**Table 1:** Selected characteristics of the respondents included in the study analyses

Variables	2000	2002	2004	2006	2008
**Continuous variables**					
Job stress	19.980	20.126	19.790	19.480	19.105
	(4.715)	(4.767)	(4.870)	(4.848)	(5.051)
Age	37.180	37.450	38.190	38.480	39.450
	(11.610)	(11.870)	(11.790)	(12.029)	(12.280)
Health utility index	0.925	0.904	0.900	0.906	0.897
	(0.129)	(0.143)	(0.151)	(0.142)	(0.146)
Chronic diseases	0.979	1.185	1.160	1.280	1.325
	(1.265)	(1.325)	(1.306)	(1.368)	(1.404)
**Categorical variables**					
**Smoking bans**					
Full ban	0.471	0.483	0.505	0.520	0.499
	(0.499)	(0.500)	(0.500)	(0.500)	(0.500)
Partial ban	0.364	0.362	0.365	0.367	0.413
	(0.481)	(0.481)	(0.481)	(0.482)	(0.493)
No ban	0.164	0.155	0.128	0.110	0.083
	(0.370)	(0.362)	(0.334)	(0.313)	(0.275)
**Gender**					
Male	0.557	0.530	0.544	0.558	0.557
	(0.497)	(0.499)	(0.498)	(0.496)	(0.497)
Female	0.442	0.469	0.455	0.442	0.442
	(0.497	(0.499)	(0.498)	(0.496)	(0.497)
**Marital status**					
Single	0.331	0.347	0.318	0.330	0.295
	(0.471)	(0.476)	(0.466)	(0.470)	(0.456)
Married	0.526	0.511	0.537	0.530	0.559
	(0.499)	(0.500)	(0.498)	(0.499)	(0.496)
Separated	0.141	0.141	0.144	0.139	0.145
	(0.349)	(0.348)	(0.351)	(0.346)	(0.352)
**Education level**					
Less secondary education	0.174	0.167	0.139	0.125	0.141
	(0.380)	(0.373)	(0.346)	(0.331)	(0.348)
Secondary education	0.183	0.175	0.171	0.152	0.141
	(0.387)	(0.380)	(0.376)	(0.359)	(0.348)
Some post secondary	0.301	0.316	0.320	0.325	0.303
	(0.459)	(0.465)	(0.466)	(0.468)	(0.459)
Post secondary	0.339	0.341	0.369	0.397	0.413
	(0.474)	(0.474)	(0.482)	(0.489)	(0.493)
**Income level**					
Low income	0.064	0.062	0.054	0.034	0.024
	(0.246)	(0.241)	(0.226)	(0.181)	(0.154)
Low middle income	0.201	0.175	0.176	0.121	0.110
	(0.401)	(0.380)	(0.381)	(0.327)	(0.313)
High middle income	0.441	0.395	0.358	0.370	0.317
	(0.497)	(0.489)	(0.480)	(0.483)	(0.466)
High income	0.294	0.368	0.411	0.475	0.548
	(0.456)	(0.482)	(0.492)	(0.500)	(0.498)

The statistics are weighted using the NPHS sampling weights. The sample is restricted to working smokers at each wave of the study period. For categorical variables, the mean represents the proportion of each subgroup in its relevant category while the corresponding standard errors are in parenthesis. Data source: Statistics Canada NPHS, wave 4(2000) to wave 8(2008)

## Methods

To examine the relationship between WSB's and self-perceived, work-related stress, a fixed-effects linear regression is used in order to control for measured and unmeasured individual time-invariant confounders. The use of the fixed-effects method is appropriate, given that some crucial unmeasured individual factors (e.g. cognitive abilities) can bias the outcome between WSB's and workplace stress.

A fixed-effect regression model of the following form is estimated:

$$workstress_{ijt}=\beta_{0}+\beta_{1}ban_{ijt}+\beta_{2}X_{ijt}+\mu_{i}+\varepsilon_{ijt}$$

where *i, j and t *represent, respectively, an individual, province of residence and time period. The symbol *ban *represents the three categories of WSB's, × is a vector of additional control variables which include: age, marital status, educational attainment, ethnicity, work status, income, drinking status, health status, number of chronic conditions, provincial and occupational fixed effects. Individual time-invariant characteristics are represented by μ_i _and can be correlated with other time-varying covariates in the model. The standard residual term is, *ε*_*ijt *_which is adjusted for clustering at the individual level. In order to study the heterogeneous effects of workplace smoke-free policies on self-perceived stress, separate analyses are performed for males, females, young adult (age 18-40 years) and those classified as old (age 41-65 years). To check whether the results are sensitive to the inclusion of additional covariates, three different model specifications are estimated. Model 1 is the baseline specification which controls for income level, educational level, marital status, age, work status, ethnicity, drinking status. Model 2 includes the additional covariates: individual health status, number of chronic conditions and provincial fixed effects. In model 3, occupational fixed effects are included, in addition to the model 2 covariates. Also, as a further robustness, a separate analysis is performed for non-smokers. Multivariate analysis is conducted using Stata 11.

## Results

### Descriptive statistics

The sample characteristics of working smokers are presented in Table 1. The mean of the Work Stress Index is above 19 in each wave. About 47% of the sample in 2000/01 reported a complete smoke-free policy in their workplaces, while 36% and 16% have a partial smoking ban and no ban respectively. Workplace with no smoking restriction has experienced a considerable decrease over the study period. For example, the percentage of working smokers who reported no smoking restrictions in their workplaces decreased by about 49% percent from 2000 (16.4%) to 2008 (8.3%). The gender composition at each wave of the study period indicates that there are more males than females in the sample. A significant proportion of the sample is well educated, with majority having completed at least some post-secondary education.

### Regression results

The results from the fixed effect linear regression along with the 95% confidence intervals (CI's) are reported in Table 2. The results for the whole sample show that WSB's have a positive and statistically significant association with self-perceived work stress. In particular, on average, smokers in jobs with complete WSB's report a higher work stress level *(β = 0.75, CI = 0.19-1.32) *compared to those with no workplace smoking restriction. Also, smokers with partial workplace smoking restrictions report higher work stress *(β = 0.69, CI = 0.12-1.26) *compared to the reference category (no workplace smoking restriction). Similar results are obtained in models 2 and 3 that the association between WSB's and self-reported work stress is positive and statistically significant.

**Table 2:** Fixed-effect regression estimates of the effects of workplace smoking bans on work-related stress among smokers

	Model 1	Model 2	Model 3
**Whole sample**			
Full ban	0.752***	0.726**	0.783***
	(0.188-1.316)	(0.162-1.291)	(0.225-1.340)
Partial ban	0.688**	0.668**	0.704**
	(0.117-1.260)	(0.096-1.240)	(0.138-1.270)
*Observations*	7763	7763	7763
**Males**			
Full ban	0.905***	0.848***	0.918***
	(0.270-1.540)	(0.211-1.484)	(0.284-1.553)
Partial ban	0.779**	0.725**	0.739**
	(0.130-1.428)	(0.079-1.370)	(0.086-1.391)
*Observations*	4071	4071	4071
**Females**			
Full ban	0.402	0.377	0.396
	(-0.864-1.669)	(-0.903-1.657)	(-0.853-1.645)
Partial ban	0.411	0.416	0.471
	(-0.880-1.702)	(-0.887-1.719)	(-0.809-1.751)
*Observations*	3692	3692	3692
**Age 18-40**			
Full ban	0.978**	0.975**	1.106**
	(0.086-1.869)	(0.081-1.869)	(0.236-1.976)
Partial ban	0.888**	0.900**	0.992**
	(0.012-1.765)	(0.018-1.781)	(0.143-1.840)
*Observations*	4055	4055	4055
**Age 41-65**			
Full ban	0.349	0.320	0.378
	(-0.380-1.078)	(-0.412-1.052)	(-0.366-1.121)
Partial ban	0.275	0.241	0.290
	(-0.490-1.040)	(-0.526-1.008)	(-0.481-1.061)
*Observations*	3708	3708	3708

Confidence intervals are in parentheses.*** *p *< 0.01, ** *p *< 0.05, * *p *< 0.1. Model 1 is the baseline specification which controls for income level, educational level, marital status, age, work status, ethnicity, drinking status. While model 2 includes additional covariates: individual's health status, number of chronic conditions and provincial fixed effects. In addition to model 2, occupational fixed effects are included in model 3. All estimations are weighted using the sample weight available in the NPHS. Data source: Statistics Canada NPHS, wave 4(2000) to wave 8(2008)

The regression results from sample splitting (by gender and age) reveal that the relationship between WSB's and work stress may vary across different groups. For males, a complete workplace smoking restriction increases work stress *(β = 0.92, CI = 0.28-1.55) *among smokers compared to those with no workplace smoking restriction. Similarly, partial workplace smoking restriction increases work stress *(β = 0.74, CI = 0.09-1.39) *compared to the reference category. While the effect of WSB's on work-related stress is also positive for females, the estimates are not statistically significant. For example, in Table 2 a full WSB increases work stress *(β = 0.40, CI = -0.85-1.65) *compared to no workplace smoking restriction. This result is not statistically significant as the CI includes zero. The results by age group show that WSB's have a statistically significant effect on work stress for those aged 18-40 years (young adult) but there is no effect on those of 41-65 years. Compared to those in no WSB, full WSB increases work stress *(β = 1.11, CI = 0.24-1.98) *for young adult smokers and *(β = 0.38, CI = -0.37-1.21) *for old smokers. To check whether the association between WSB's and work-related stress varies by smoking status, a separate analysis is undertaken for non-smoking workers. Results for the non-smokers, which are presented in Table 3, show that WSB's have no statistically significant association with self-perceived work stress. This is not surprising, given that WSB's may be a redundant restriction for non-smokers.

**Table 3:** Fixed-effect regression estimates of the effects of workplace smoking bans on work-related stress among non-smokers

	Model 1	Model 2	Model 3
**Whole sample**			
Full ban	-0.057	-0.056	-0.035
	(-0.434-0.320)	(-0.435-0.323)	(-0.412-0.342)
Partial ban	-0.023	-0.030	-0.025
	(-0.418-0.371)	(-0.427-0.366)	(-0.420-0.370)
*Observations*	21345	21345	21345
**Males**			
Full ban	0.032	0.029	0.072
	(-0.439-0.503)	(-0.445-0.504)	(-0.399-0.543)
Partial ban	0.011	-0.002	0.021
	(-0.471-0.494)	(-0.488-0.484)	(-0.460-0.504)
*Observations*	10256	10256	10256
**Females**			
Full ban	-0.201	-0.189	-0.188
	(-0.799-0.397)	(-0.785-0.408)	(-0.787-0.410)
Partial ban	-0.105	-0.091	-0.103
	(-0.748-0.539)	(-0.731-0.549)	(-0.745-0.539)
*Observations*	11089	11089	11089
**Age 18-40**			
Full ban	-0.226	-0.199	-0.222
	(-0.868-0.415)	(-0.847-0.449)	(-0.872-0.428)
Partial ban	-0.199	-0.209	-0.246
	(-0.870-0.471)	(-0.886-0.468)	(-0.923-0.431)
*Observations*	9557	9557	9557
**Age 41-65**			
Full ban	-0.131	-0.137	-0.107
	(-0.595-0.334)	(-0.603-0.329)	(-0.573-0.359)
Partial ban	-0.027	-0.036	-0.014
	(-0.510-0.455)	(-0.520-0.446)	(-0.497-0.468)
*Observations*	11788	11788	11788

Confidence intervals are in parentheses. *** *p *< 0.01, ** *p *< 0.05, * *p *< 0.1. Model 1 is the baseline specification which controls for income level, educational level, marital status, age, work status, ethnicity, drinking status. While model 2 includes additional covariates: individual's health status, number of chronic conditions and provincial fixed effects. In addition to model 2, occupational fixed effects are included in model 3. All estimations are weighted using the sample weight available in the NPHS. Data source: Statistics Canada NPHS, wave 4(2000) to wave 8(2008)

## Discussion and Conclusion

The adverse health consequences of environmental tobacco smoke are well established. In response, smoking bans have been progressively used in recent times. Besides protecting non-smokers against environmental tobacco smoke, some studies show that WSB's reduce smoking prevalence and intensity. While there is substantial literature on the effect of this policy in altering smoking behaviors, little is known about its potential unintended consequences. This study uses longitudinal data from the Canadian National Population Health Survey to examine the association between WSB's and work-related stress. We exploit the panel structure of the data set by using a linear fixed-effects model to control for individual time-invariant confounders that may bias the relationship between WSB's and work-related stress. To capture the heterogeneous effect of WSB's, on stress level across different groups of workers, the analysis is stratified by gender and age.

Multivariate analysis reveals that, on average, full or partial WSB's have a positive and statistically significant association with self-perceived work-related stress among smoking workers. These findings are not sensitive to the inclusion of individual's health status, occupational and provincial fixed effects. We also find that these effects vary by gender and age. In particular, WSB's are significantly associated with higher work stress for males and young adults (aged 18-40). We also examined the effect of WSB's on the stress level of nonsmoking workers and no statistically significant effect was found (see Table 3).

These results are supported by some previous studies which find that WSB's have a larger impact on groups with high smoking rates [27-29]. For example, Heloma and Jaakola [22] find that Finland's national smoke-free workplace laws have a greater impact on males than on females. In a recent survey of 2,103 workers by Croner [30], two thirds of the workers reported that they would not manage their stress very well, or at all, if there were a ban on smoking breaks.

These individual differences in the effect of WSB's on stress level could be explained by the varying degree of nicotine dependence and the smoking rate among individuals. Smokers with low nicotine dependence are less likely to suffer from symptoms of nicotine withdrawal, including tension and mood disturbance [31]. This may help explain why there are gender differences in the results of the present study. Some studies show that females are less nicotine dependent than males [e.g. [32]]. Variations in nicotine dependence have been attributed to some unobserved characteristics, including genetic factors, personality and family background [31].

The results of the current study could imply additional negative effects of higher smoking intensity, given several studies find that work stress is positively associated with more smoking [e.g. [33-37]]. Also, the health risks and economic costs attributable to job stress are substantial and widely documented [38,39]. For example, work stress costs U.S. companies over $300 billion annually [40] and has been linked to several adverse health conditions [41].

In addition to increased level of stress among smokers, other unintended consequences of WSB's have been reported in previous studies. WSB's may lead to compensating smoking behaviors [8,9]. For example, some smokers may increase cigarette consumption before and after work [42], smoke hard during break times by increasing puff frequency and fast smoke each cigarette [8]. Other side effects include smoking in unsafe places and the accumulation of smoking trash like cigarette butts and dead matches at the entrance to a workplace. Also, the congregation of smokers at the entrances and exits of a workplace increases exposure to second-hand smoking.

The current study has some limitations. First, we did not control for individuals' level of nicotine dependence as this information is not available in the data set. Second, the fixed-effects model that we used controls only for individual time-invariant confounders. However, there may be other time-variant unobserved characteristics that are not captured by the current study. Third, it is possible that differences in work-related stress levels among individuals may be due to differences in the type of workplace and not the smoking bans, especially if these bans are constant over time. However, the analysis uses seven occupational dummies to control for unobserved occupation-specific characteristics that may affect stress level. It should be noted that these occupational dummies may not fully capture individual-specific workplace effects, since individual work environments within each occupational classification are likely to be different. Fourth, this study does not stratify the analysis by quantity of cigarettes smoked. Recent studies cast doubt on using the individual quantity of cigarettes smoked as a measure of smoking intensity, where smokers may reduce the quantity of cigarettes smoked but increase the intake of cotinine [43,44]. The NPHS data set does not have any information on cotinine. Also, there is no uniform definition on the number of cigarettes smoked that can be classified as heavy or light.

Despite these limitations, this paper adds to the literature on the unintended consequences of anti-smoking policies by providing empirical evidence that WSB's may lead to higher self-perceived, workplace-related stress among smoking workers. These results cannot be generalized. Further evidence using data from other countries will be necessary to confirm these findings. The unintended negative side effects of WSB's do not undermine their usefulness in discouraging smoking. However, it is important for policy makers to combine workplace smoke-free policies with other workplace intervention measures, in order to offset the negative side effects. For example, stress management programs [41], individual and group therapy and pharmacological treatment to address nicotine addiction have been shown to be effective intervention strategies [7]. Moreover, it may also be useful to complement WSB with other worksite-based tobacco control intervention measures to promote smoking cessation among workers [45]. The results of this study do not imply that WSB's are the main determinant of self-perceived, work-related stress among smokers but provides suggestive evidence that they may be positively related.

## Competing interests

The authors declare that they have no competing interests. This study is not funded by any organization including Tobacco Companies or its affiliates.

## Authors' contributions

Design of the study: SA, MFS. Data analysis: SA. Writing of manuscript: SA, MFS. Both authors read and approved the final manuscript.

## Pre-publication history

The pre-publication history for this paper can be accessed here:

http://www.biomedcentral.com/1471-2458/12/123/prepub
